# Performance and tolerance of *Moringa stenopetala* exposed to drought stress during germination and growth

**DOI:** 10.1371/journal.pone.0329544

**Published:** 2025-11-03

**Authors:** Nadia Khater, Kenza Garah, Toufik Aliat, Nyasha J. Kavhiza, Hani S. Saudy, Karim M. Hassan, Mahmoud A. A. Ali, Mostafa Abdelkader

**Affiliations:** 1 Department of Ecology and Environment, Faculty of natural and life sciences, University of Batna, Batna, Algeria; 2 Biodiversity, Biotechnology, and Sustainable Development Research Laboratory, Batna, Algeria; 3 Laboratory of Algerian Forests and Climate Change, Higher National School of Forests, Khenchela, Algeria; 4 Department of Environmental Management, Institute of Environmental Engineering, RUDN University, Moscow, Russia; 5 Agronomy Department, Faculty of Agriculture, Ain Shams University, Cairo, Egypt; 6 Horticulture Department, Faculty of Agriculture, Ain Shams University, Cairo, Egypt; 7 Horticulture Department, Faculty of Agriculture, Sohag University, Sohag, Egypt; Forest Survey of India, INDIA

## Abstract

*Moringa stenopetala* is a rapidly growing, unappreciated tree regarded as the “miracle tree” for its food, feed, and medicinal benefits. It appears to be a versatile and promising species for use under changing conditions. However, the biological response of Moringa stenopetala, a valuable but underutilized species, to drought remains unclear during its early growth stages. Therefore, the main aim of this study was to investigate the morphological, physiological, and biochemical responses of *M. stenopetala* seedlings to different levels of drought stress induced by PEG-6000. Four concentrations of PEG-6000 (0, 4, 8, and 12 percent) were applied to evaluate the effect of water deficit on the morphological, physiological, and biochemical characteristics of *M. stenopetala*. The results indicated that the water potential exhibited a statistically significant impact on the germination rate (82.5%) and the mean germination time. The osmotic potential of the PEG solution was found to have a significant impact on germination speed (93%), the kinetics of germination (39%), the germination index (102%), and the germination vigour index (91.25). The study further revealed a positive correlation between water stress and increased stem growth and root length. Concurrently, proline content exhibited a substantial decrease, directly proportional to the stress level incurred. The accumulation of soluble sugars in the leaves exhibited variation according to stress levels. Drought indices revealed that the moderate treatment of PEG gave the highest GSI (1.325), STI (1.325), MPI (0.93), and HM (0.906), indicating drought tolerance and potential regular growth under drought stress. PCA visualized the most relationships among the studied traits of Moringa under drought stress, revealing distinct groupings and key indicators of drought tolerance, where the first two principal components (PC1 and PC2) explain most data variation (83%). These conclusions emphasize the adaptive ability of Moringa under drought stress conditions, besides proving its application as a drought-resilient crop.

## 1. Introduction

Climate change leads to severe abiotic stress conditions, causing physio-biochemical disorders significantly reducing growth and develoment of the crops [1–5]. Abiotic stressors such as drought, heat, and salinity adversly impact plant growth and productivity [6–12]. Drought stress is a worldwide problem, and a major part of arable land is affected by various degrees of drought [13–18]. Drought stress represents one of the plants’ most impactful forms of environmental stress [19–23]. Insufficient water supply to meet the plant’s needs, whether due to dry soil or dry weather conditions, constitutes drought stress [24–27]. The degree of water stress experienced by a plant is dependent on several factors, including the capacity of the plant to absorb water from the soil, the loss of water by the green parts of the plant through processes of evaporation and photosynthesis, and the tolerance of the plant to drought conditions [28–31]. It is widely acknowledged that environmental stress, particularly drought stress, significantly inhibits plant development and productivity [32–35].

Moisture is crucial for seeds to absorb water and begin germination [36]. To simulate drought conditions under low water availability, researchers often use osmotic agents to study germination responses [37]. Drought can negatively impact plant regeneration, growth, and survival [38] because the reduced osmotic potential in dehydrated seeds restricts metabolic activity [37]. Among abiotic stresses, water availability is the most critical factor influencing seed germination and seedling establishment [39]. The severity of drought effects varies across species, as different plants have developed diverse strategies to cope with limited water [40,41]. Evaluating drought tolerance at the germination stage provides key insights into species survival, community dynamics, and potential responses to climate change [42].

In order to maintain a high water level and resist dehydration during a drought, the plant employs many adaptive strategies [43–45]. In order to conserve water, plants close their stomata, produce substances that enable them to withstand drought conditions, and adjust the growth of their roots to facilitate the extraction of soil water and nutrients [46–50]. Nevertheless, prolonged exposure to this stress can harm the plant’s health, reducing growth, productivity, and resistance to diseases and pests [51]. Plants have developed a variety of adaptive strategies to survive environmental challenges [52–54]. During drought conditions, they regulate how they use water, enhance their ability to draw moisture from the soil, and build stronger drought resistance through genetic variation [55,56].

It is important to note, however, that prolonged water stress can adversely affect plant health and survival. Adapting plant species that are both highly beneficial to humanity and resistant to abiotic stresses represents a strategy to restore biodiversity in arid and semi-arid regions [57–59]. Tropical areas are home to plants in the Moringaceae family, including Moringa [60], that includes 13 species native to India, Red Sea, and Madagascar [61]. This versatile tree finds extensive applications across industry, agriculture, and medicine, thereby serving as a critical element of the economic infrastructure of civilizations.

Moringa is distinguished by its ability to produce flowers even under conditions of limited water availability, thus demonstrating its adaptability to water-scarce environments. This capacity results from several physiological and morphological adaptations [62]. For example, Moringa leaves are equipped with stomata, which are microscopic openings that regulate gas exchange and sweating. During periods of water stress, Moringa can partially close its stomata to mitigate water loss through transpiration, thus sustaining its internal moisture balance [63]. The tree species Moringa stenopetala, indigenous to East Africa, has attracted considerable attention due to its exceptional drought tolerance, high nutritional value, and potential applications in food, medicine, and environmental conservation [64,65].

This research is expected to enhance the understanding of plant drought tolerance and offer insights into optimizing the cultivation of M. stenopetala in water-limited environments. By analyzing germination parameters and early stages of growth and development of seedlings under different levels of osmotic stress induced by PEG-6000, this study aims to elucidate the response of M. stenopetala seeds to water deficit conditions.

## 2. Materials and methods

This study was conducted at the Laboratory of Ecophysiology, Department of Ecology and Environment, Faculty of Natural and Life Sciences, University of Batna 2. Moringa seeds were collected from a forest conservatory nursery for the Wilaya of Oued Souf in southeast Algeria.

### 2.1. Seed preparation and experimental approach

Seeds were washed with tap water for 5 min, and then they were immersed in a solution of sodium hypochlorite (10%) for 15 minutes and washed in sterile water three times to remove any leftover sodium hypochlorite. The disinfected seeds were placed in plastic boxes (19.5 cm in length and 13.5 cm in width) lined with three layers of filter paper saturated with distilled water at a rate of 8 seeds per box, with five repetitions per treatment (40 seeds per treatment). The absorbent paper is soaked with the PEG-6000 solution from each treatment. Four different concentrations of PEG-6000 (Control, PEG4%, PEG8%, and PEG12%) were applied. The boxes were placed in the dark on a stove at 25 °C and soaked with 15 ml of different treatments. The absorbent paper is soaked with the PEG-6000 solution from each treatment.

The appearance of a visible 2 mm radicle was considered the germination point for the seeds. Regular daily observations of the experiment were carried out. For a maximum of one week, data were collected once every day. The germinated seeds were cultivated in pots containing a mixture of two-thirds commercial potting soil and one-third sand, with one seed per pot and ten repetitions in a controlled growth chamber with a temperature of 25°C and a 16-hour photoperiod. Watering was done daily, with each plant receiving 5 ml of water, once with the treatments and once with tap water.

### 2.2. Germination measurements

#### 2.2.1. Germination rate.

The germination rate (GR) was calculated according to the following formula [66]:.


TG (%) = Gx/Gt × 100

Where is TG: Final germination rate, Gx: Number of germinated seeds, and Gt: Total number of seeds sown

#### 2.2.2. Germination speed.

It is expressed by the germination rate obtained at a given time:.


100 (N1+N2+⋯+ Nn)/([N1T1+N2T2+⋯+ NnTn])%

Where is N1: number of seeds germinated at the time T1, N2: number of seeds germinated between time T1 and T2, and Nn: seeds germinated at time T3… Through time

#### 2.2.3. Mean Germination Time (MGT).

The mean germination time was calculated [67] as follows:.


MGT = ΣNiTi/ΣNi

Where Ni: Number of seeds newly germinated at time Ti, Ni + 1: Number of seeds germinated between Ti and Ti + 1

#### 2.2.4. Germination index.

The germination index was calculated [68] as follows:.


GI= (7×N1) + (6×N2) + …1 = Nn (//…)

Where GI—Germination index, N1, N2 …, Nn—Number of seeds germinated over 1, 2, …, n days of germination.

Multipliers (7, 6, …)—Weights assigned to germination days.

#### 2.2.5. Germination vigour index.

Germination index was calculated [69] as follows:.


IVG = (a/1 + b/2 + c/3… +z/n) × 100/S

Where a, b, c, …, z are the numbers of seeds germinating each day; n: the number of days the experiment lasts (8 days in our case); S: the number of seeds tested (20 seeds in our case).

#### 2.2.6. Germination kinetics.

To better learn the physiological significance of the germinative behaviour of the varieties studied, the number of germinated seeds was counted daily until the seventh day of the experiment [70].

### 2.3. Biochemical parameters

#### 2.3.1. Chlorophyll content.

Chlorophyll a and b were determined using the methodology developed by Nagata and Yamashta [71]. One gram of moringa leaf was crushed in 10 ml of 80% acetone and filtered through Whatman No. 1 filter paper. The filtered extract was transferred to a cuvette. Absorbance was measured at 663. 645. 505 and 453 nm using a UV-spectrophotometer (UV-4000, ORI Germany). The following formulas were used to calculate chlorophyll a and chlorophyll b concentrations [72].


Chlorophyll a = 0.999 A663 − 0.0989 A645

Chlorophyll b = −0.328 A663 + 1.77 A645

#### 2.3.2. Determination of Proline and Sugar Contents.

The free proline content was measured using the ninhydrin method [73]. The soluble sugar content was determined colourimetrically using phenol sulphuric acid [74,75].

#### 2.3.3. Determination of total free amino acids.

100 mg of dry matter (DM) leaves and roots are ground separately in 1 ml of distilled water and then placed in a boiling water bath (100°C) for one hour. The mixture is then centrifuged at 5000 rpm for 10 min. The pellet is subjected to a second extraction with 1 ml of ethanol. 200 µL of the extract is mixed with 500 µL of citrate buffer. 1 ml of the ninhydrin-ascorbic acid reaction mixture is added to the ground material. A spectrophotometer measured the total amino acid content expressed in mg/g^-1^ DM at a wavelength of 570 nm [76,77]..

#### 2.3.4. Drought Indices.

Drought indices were employed to measure the drought tolerance of moringa seedlings by comparing nutrient and water accumulation under various drought stress conditions with those under non-stressed conditions. These indices quantify drought tolerance based on growth reduction under stress, helping to identify treatments with stable performance. Total weight (shoots and roots) was recorded for each treatment, and the seedlings not treated with PEG (control) were designated as the non-stressed control. The data were used to compute various drought indices described in previous studies [78,79]. These indices provided a complete vision of the performance and stability of moringa under water-deficit conditions, facilitating the identification of drought-tolerant treatments. The indices were calculated according to the following equations:.


GSI=Ys/Yp;

TOL=Yp−Ys; 

 MPI=0.5×(Ys +Yp);

STI=(Ys×Yp)/(Yp);

HM=(2×Ys×Yp)/(Ys+Yp)

Where: GSI = Growth Stability Index; TOL = Tolerance; MPI = Mean Productivity Index; STI = Stress Tolerance Index

HM = Harmonic Mean; Ys = accumulated weight under drought stress; Yp = accumulated weight under non-stress (control) conditions.

Drought indices are valuable tools for assessing the tolerance of moringa seedlings by comparing their growth under stressed and non-stressed conditions. They help measure yield reduction under drought and identify genotypes with stable performance. The Growth Stress Index (GSI) shows the proportion of growth maintained under stress, while the Tolerance Index (TOL) measures yield loss, with lower values indicating stronger resistance. The Mean Productivity Index (MPI) reflects average performance across both environments, and the Stress Tolerance Index (STI) highlights plants that combine good productivity with drought tolerance. The Harmonic Mean Index (HM) emphasizes consistent performance by giving more weight to the lower yield, making it especially useful for identifying best applied treatment. Together, these indices provide a comprehensive evaluation of plant resilience under water deficit conditions.

### 2.4. Statistical analyses

The experiment was designed using a completely randomized design (CRD) with five replicates. A one-way analysis of variance (ANOVA) was applied to determine differences among the studied treatments. The data were analyzed using the SPSS software, V. 22.30 bit, presented as mean ± SD, and compared using the Tukey test (p < 0.05). Principal Component Analysis (PCA) was perfurmed using R programming V. 24.12.1

## 3. Results

### 3.1. Germination indices

**Fig 1:**
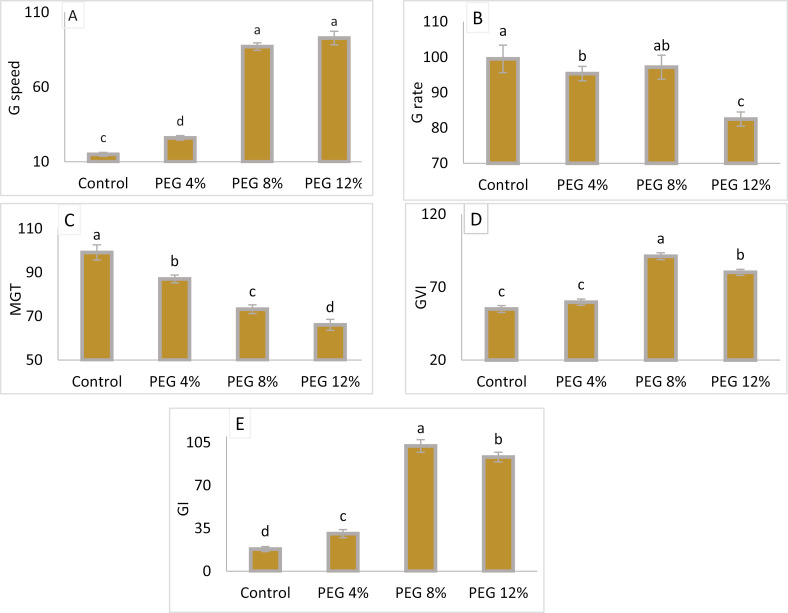
Germination speed(A), Germination rate (B), Mean germination Time(MGT) (C), Germination Vigor Index (GVI) (D), and Germination index(GI) (E), of M. stenopetala seeds under the effect of different PEG concentrations. Means showing different letters are statistically different (p < 0.05).

Germination indices, i.e., germination rate, germination speed, mean germination time, germination index and germination vigour index as infllunced by different PEG-6000 treatments are illustrated in Fig 1 (See the original data in S1 Table).

#### 3.1.1. Germination rate.

Increasing PEG concentrations significantly decreased the germination rate (P < 0.05). It was noted that the highest germination rate was recorded in the treatments PEG4% and PEG8% after control, reaching 97% and 95%, respectively, while the lowest rate of 82.5% was recorded in the PEG12% treatment.

#### 3.1.2. Germination speed.

Effects of different PEG-6000 treatments (P < 0.05) on germination speed were significant. Treatment PEG12% had the fastest germination rate, estimated at 93%, whereas treatment PEG4% had the slowest, at around 15%, followed by the control.

#### 3.1.3. Mean germination time.

The results demonstrate that MGT significantly decreases (P < 0.05) with increasing PEG concentrations. High MGT values were recorded at 84 hours for seeds treated with PEG4% after control compared to those treated with PEG8% and PEG12%, which had 72 and 67 hours, respectively.

#### 3.1.4. Germination index.

The results demonstrate a significant progressive increase in the seeds’ vigor index with PEG concentration increase. The highest value was recorded at 102 in treatment PEG8%, followed by PEG12%, reaching 93, while the lowest values were estimated at 30 in PEG4%, followed by the control treatment..

#### 3.1.5. Germination vigour index.

Effects of different PEG-6000 treatments (P < 0.05) on the germination vigor index of M. stenopetala were non-significant. The highest value of 91.25 was observed in PEG12%, followed by 80 in T4, and the lowest value of 55 recorded in PEG4% followed by control.

#### 3.1.6. Germination kinetics.

**Fig 2:**
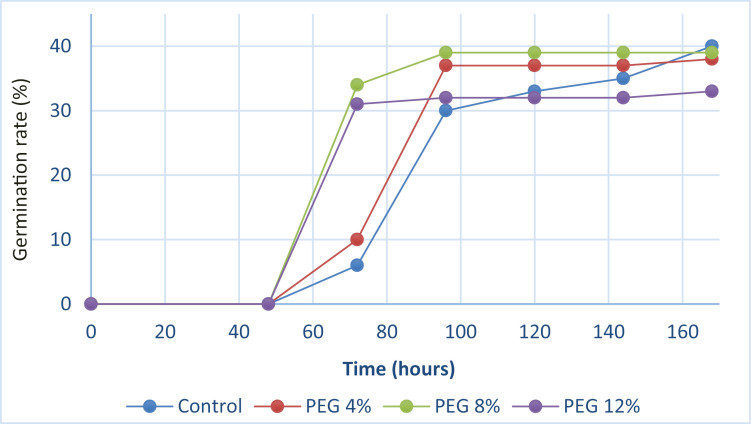
Seed germination kinetics of M. stenopetala under the effect of different PEG concentrations.

The curves of germination kinetics under the effect of different PEG concentrations show a sigmoidal shape with three stages (Fig 2). A latency period is required for the appearance of the initial germination, during which the germination rate is low. The duration of this phase varies according to the concentration of PEG; for seeds treated with control, PEG4%, PEG8%, and PEG12%, it lasts for 48 hours. A second exponential phase occurs when the germination rate rapidly increases and evolves correspondingly with time. For seeds treated with PEG8% and PEG12%, the second phase lasts 24 hours. For seeds treated with control and PEG4%, the second phase lasts 48 hours.

A third phase corresponds to a plateau representing the final percentage of germination and reflects the seeds’ germination potential under the impact of various PEG concentrations. The seeds’ germination capacity appears to be lower in those treated with PEG4% and PEG12% than in those treated with control and PEG8%. As the concentration of PEG increases, the form of this curve changes in the direction of expansion, resulting in a faster germination rate (Fig 2).

### 3.2. Growth parameters

**Fig 3:**
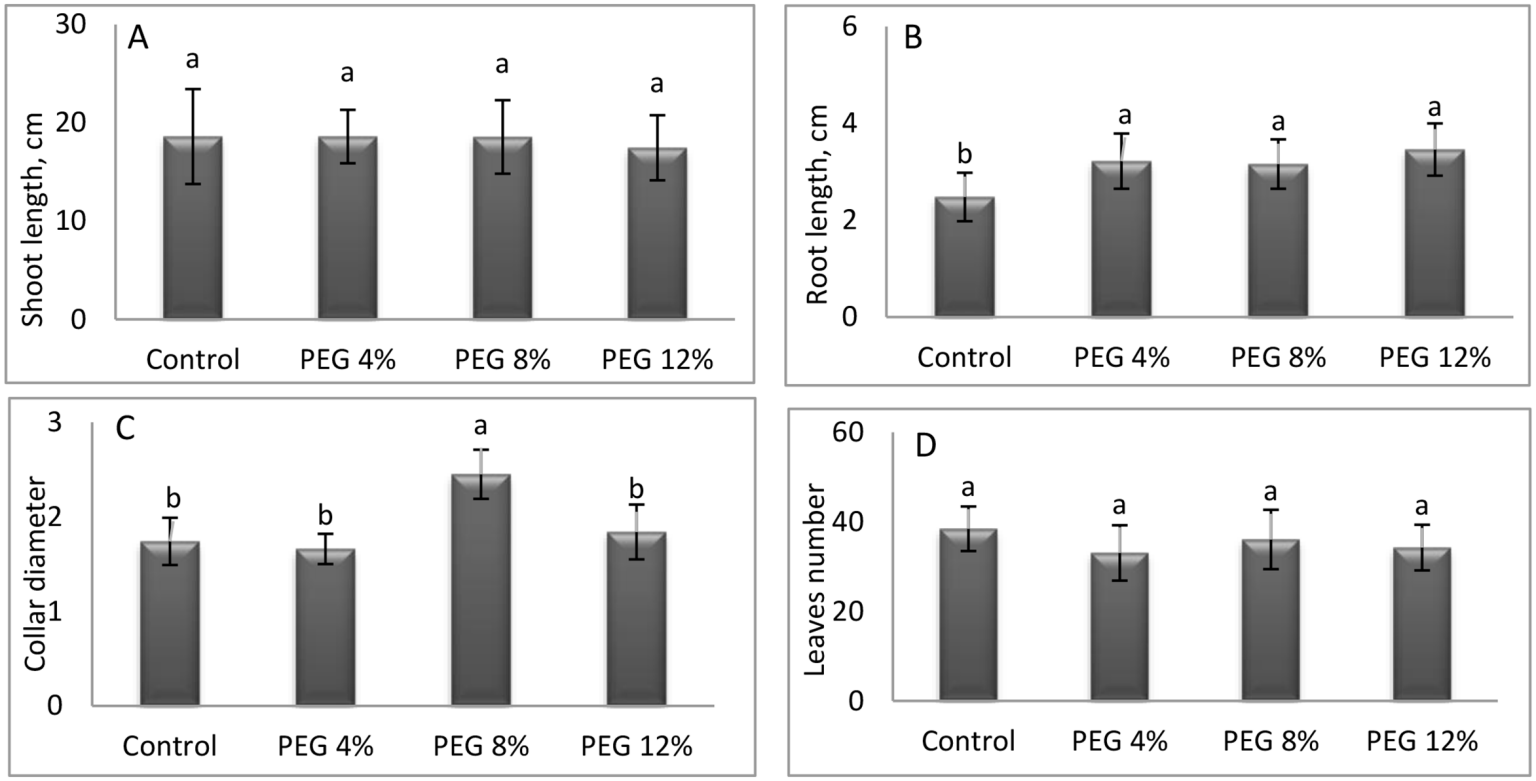
Changes in growth characteristics (A = shoot length; B = root length; C = collar diameter; D = number of leaves) of M. stenopetala under drought conditions. Means showing different letters are statistically different (p < 0.05).

The results demonstrate a substantial variation in the growth of M. stenopetala, attributed to the impact of water stress induced by increasing concentrations of PEG (See the original data in S1 Table). The number of leaves exhibited a significant variation in response to varying concentrations of PEG, with the highest recorded value of 36.06 observed in PEG8% following the control treatment and a lower value recorded in PEG4% (Fig 3)

**Fig 4:**
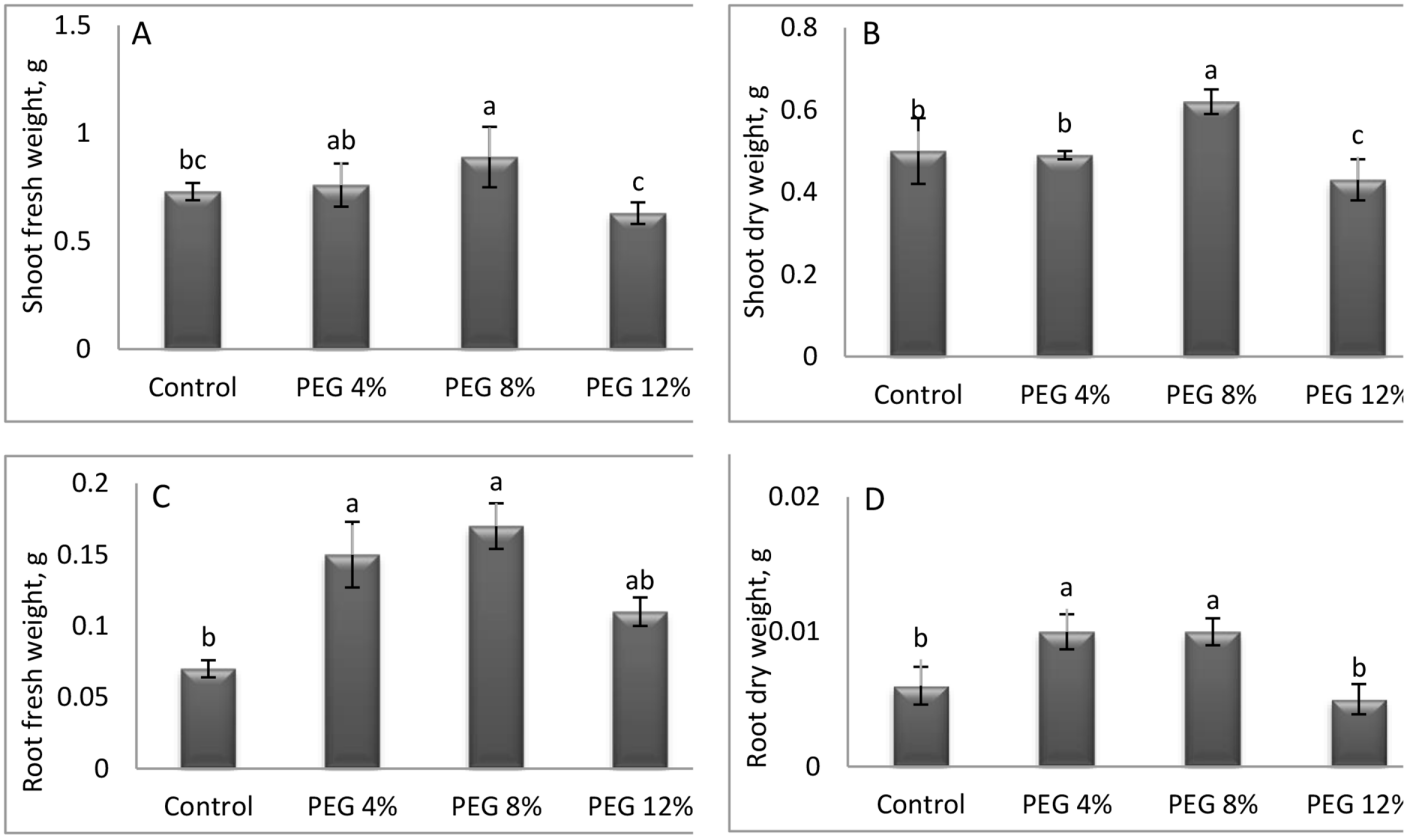
Changes in growth characteristics (A = shoot fresh weight; B = shoot dry weight; C = root fresh weight; D = root dry weight) of M. stenopetala under drought conditions. Means showing different letters are statistically different (p < 0.05).

Concerning the shoot length (SL), the least significant deviation was observed in the PEG12% treatment, with a recorded value of 17.46 cm. Conversely, the highest recorded values were observed in the control and PEG4% treatments, measuring 18.6 cm. Consequently, this parameter exhibited no substantial response to water stress. The study concluded that SL remained unaltered by the various treatments applied.In contrast, a significant response was observed in root length (RL), with PEG12% exhibiting the highest value (3.46 cm) and control treatment showing the lowest (2.48 cm). This method can enhance soil investigation and resource use in dry conditions. In PEG8%, the collar diameter (CD) reached its maximum at 2.45 cm, indicating optimal structural growth at moderate stress levels. Conversely, the CD recorded its lowest value of 1.74 cm under control conditions, suggesting that growth was less pronounced under non-stress conditions. The leaf number (LN) exhibited a significant decrease in response to increasing stress levels, reaching a minimal value of 34.26 at PEG12% and a maximum value of 38.46 was obtainedfrom the control treatment. This reduction is likely an adaptive mechanism that reduces transpiration to conserve water. The maximum shoot fresh and dry weight and root fresh and dry weight were recorded under PEG8% (Fig 4). These findings suggest that moderate stress promotes optimal aerial growth.

### 3.3. Chlorophylls

**Fig 5:**
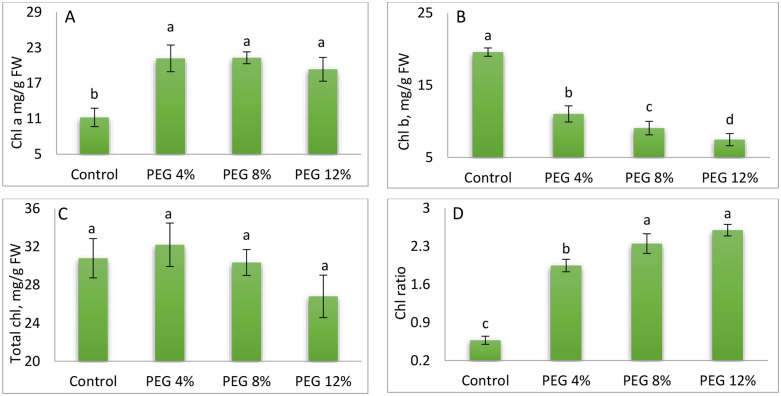
Chlorophyll a content (A), Chlorophyll b content (B), total Chlorophyll content(C), and Chlorophyll ratio (D) of M. stenopetala leaves under the effect of different PEG concentrations. Means showing different letters are statistically different (p < 0.05).

As illustrated in Fig 5(5A) which generated from the original data (S1-Table), chlorophyll a concentration in M. stenopetala presented a general increasing trend with increasing PEG levels, reaching a maximum value of 21.73 μg/g FW in treatment PEG8%. Conversely, the control’s lowest recorded concentration (12.43 μg/g FW) was observed in the control treatment. However, chlorophyll’s content declined to the highest PEG level (PEG12%). Analysis of variance (ANOVA) indicated that the differences among treatments were highly significant (p < 0.05), highlighting the substantial impact of PEG-induced osmotic stress on chlorophyll a accumulation in M. stenopetala. As presented in Fig 5(5B), chlorophyll b content in M. stenopetala decreased progressively with increasing polyethylene glycol (PEG) concentrations. The highest value (12.41 μg/g FW) was recorded in the control treatment, while the lowest (9.06 μg/g FW) was observed in treatment PEG4%. However, analysis of variance (ANOVA) revealed that the differences in chlorophyll b content among the treatments were not statistically significant (p > 0.05), suggesting that osmotic stress induced by PEG did not significantly affect chlorophyll B accumulation in M. stenopetala under the tested conditions. Fig 5(5C) presents the total chlorophyll content of M. stenopetala plants subjected to different polyethylene glycol (PEG) treatments. The control treatment showed the highest chlorophyll content (30.76 μg/g FW), while the PEG4% treatment exhibited the lowest value (29.07 μg/g FW). Analysis of variance revealed no statistically significant differences (p > 0.05) in total chlorophyll content among the PEG treatments. Concerning chlorophyll ratio, PEG8% or PEG12% showed the maximum values Fig 5 (5D).

### 3.4. Biochemical analyses

**Fig 6:**
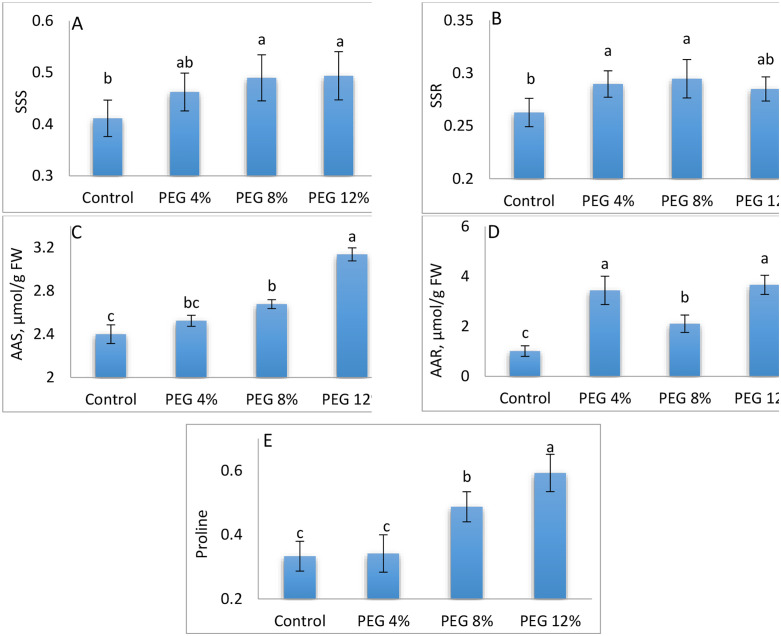
Soluble sugar content in the stem (A), Soluble sugar content in the root (B), Amino acids content in the stem (C), Amino acids content in the root (D), and proline concentration (E), of M. stenopetala seeds under the effect of different PEG concentrations. Means showing different letters are statistically different (p < 0.05).

**Fig 7:**
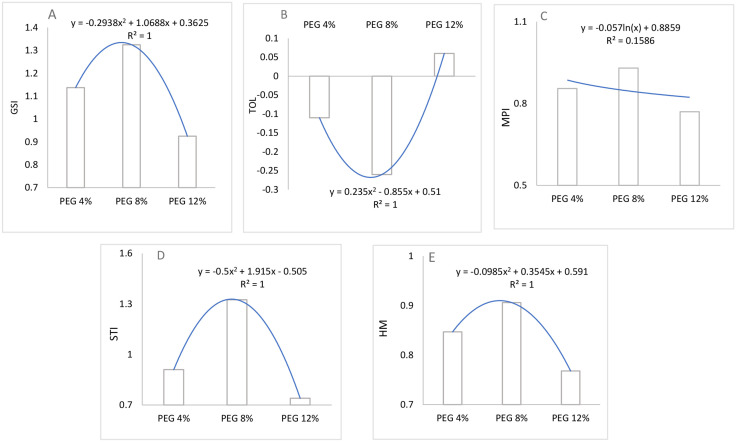
Trends of growth stability index (A), mean productivity index (B), stress tolerance index (C), and harmonic mean (D). Tolerance value (E) of moringa seedlings under different drought stress treatments (PEG4%, PEG8%, and PEG12%).

The following biochemical analyses are illustrated in Figs 6–7 which generated from S2 Table.

#### 3.4.1. Soluble sugar contents.

Fig 6 (6A and 6B) shows that the data illustrate the soluble sugar content in the aerial and root parts of M. stenopetala under different PEG treatments. The highest soluble sugar content in the aerial parts, 0.513, was recorded in the control treatment, while the lowest value, 0.462, was observed in treatment PEG4%.

In the case of root parts, the maximum soluble sugar content, 0.294, was measured in treatment PEG8%, while the minimum value, 0.262, was recorded in the control treatment. These results indicate that the soluble sugar content varies significantly between the aerial and root parts in the different PEG treatments.

#### 3.4.2. Amino acid content.

As shown in Fig 6 (6C-6D), the amino acid content in the aerial parts of M. stenopetala varied under different PEG treatments. The highest value was observed in treatment PEG12%, while the lowest was recorded in PEG4%, suggesting that PEG-induced stress affected amino acid accumulation in the aerial parts. In contrast, the amino acid content in the root system exhibited a different pattern. The highest concentration (3.661 μmol/g FW) was observed in treatment PEG12%, while the lowest (1.196 μmol/g FW) was recorded in the control. In this case, ANOVA revealed a statistically significant difference (p < 0.05) among treatments, indicating that PEG-induced osmotic stress significantly influenced amino acid accumulation in the roots of M. stenopetala.

#### 3.4.3. Proline content.

As shown in Fig 6(6E), proline concentration in M. stenopetala varied significantly under different PEG treatments. The highest proline content was observed in the control treatment, with a value of 0.593, while the lowest concentration (0.333) was recorded in treatment PEG8%. Analysis of variance (ANOVA) demonstrated significant differences (p < 0.05) in proline content among treatments, indicating that PEG-mediated osmotic stress significantly influenced proline metabolism in M. stenopetala.

### 3.5. Drought indices

The drought indices calculated from the total accumulated seedling weight of Moringa under PEG-induced stress (4%, 8%, and 12%) provided meaningful comparisons against the control group (Fig 7). The PEG 8% treatment stood out, showing the highest values in most indices such as Growth stability index (GSI = 1.325), mean productivity index (MPI = 0.93), stress tolerance index (STI = 1.325), and harmonic mean (HM = 0.906)

Interestingly, it recorded the most negative tolerance value (TOL = −0.26). These results imply that PEG 8% surpassed the control in fresh biomass yield and exhibited better stress resilience. This could be attributed to a hormetic response, where moderate stress enhances growth. The PEG 4% treatment also performed well, with all indices exceeding the control baseline, including GSI (1.1375). The data suggests that certain stress levels may trigger adaptive mechanisms in Moringa, though the exact physiological pathways remain to be explored further.

### 3.6. Principal component analysis

**Fig 8:**
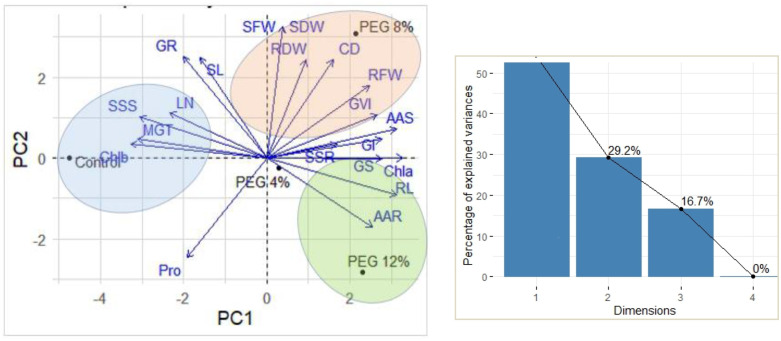
Principal component analyses (left) and cumulative components (right) of the studied parameters of moringa plants under drought stress conditions.

The PCA biplot (Fig 8) provides a multivariate summary of how early-stage Moringa plants interact with various treatments (Control, PEG 4%, PEG 8%, and PEG 12%) of drought stress conditions. The first two cumulative components (PC1 and PC2) explain most of the variation in the dataset (83.3%), offering insights into the relationships between drought intensity and key physiological and morphological traits. The positioning of the treatment points gave a clear separation with PC1, indicating a strong gradient of drought stress impact. Control and PEG4% treatments cluster closer to the left side of the biplot, indicating more moderate or baseline responses, while the highest drought treatments (PEG8% and PEG12%) shift to the far right, reflecting a significant impact of drought stress on plant growth and development. Parameters such as relative fresh weight (RFW), shoot and root dry weight (SDW, RDW), germination vigor index (GVI), and amino acid contents (AAS) align positively with higher drought levels, indicating that these parameters are more expressed or have higher variation under severe drought conditions

This may reflect Moringa’s stress adaptation strategies, such as osmotic adjustment or biomass reallocation. Meanwhile, parameters like mean germination time (MGT) and shoot length (SL) align more with control conditions, suggesting either baseline physiological levels or a reduction in these parameters under water deficit. The biplot highlights which traits are more influenced by drought and how the treatments spread in various dimensions, offering a vital framework for identifying drought-tolerant Moringa interactions and understanding stress-response mechanisms.

## 4. Discussion

Seed germination encompasses a series of metabolic processes culminating in the emergence of the radicle. This phase is crucial for establishing seedlings and plays a significant role in determining the success of agricultural production [80]. Seed germination represents a reliable and cost-effective biological indicator for assessing seed viability prior to utilization [81]. The data on germination rates demonstrated that the presence of the PEG has a highly significant impact on the germination rate of M. stenopetala seeds, particularly in the context of high levels of applied stress. The lowest value was recorded at PEG12% concentration, while the highest was observed at PEG4% after the non-stressed treatment. These findings may be related to the lower permeability of water through the seed coat and the initial absorption of water by seeds under stress conditions [82–84]. The reduction in the germination rate of M. stenopetala seeds can be attributed to the increase in various concentrations of PEG, which diminishes the water potential gradient between the seeds and their surroundings. Such water stress can deleteriously affect seeds’ normal growth and development processes, including a reduction in water and nutrient absorption, a decline in enzymatic activity, and alterations in cellular structure and function. Consequently, this adverse impact on the growth rate of seeds germinated at varying levels of PEG concentration. Therefore, these findings are consistent with another stufy on M. oleifera, which demonstrates that the presence of PEG is an osmotic agent in the drying of seeds [85].

Analysis of the germination rate results shows that different PEG treatments accelerate germination and increase the germination capacity of M. stenopetala seeds. It was observed that when the PEG concentration is high, the seed germination rate increases. This is because PEG creates a water tension that limits drought conditions. It is known that root stimulation improves the germination process and leads to faster germination of seeds [86] as the embryonic period is slow, the seed germination rate becomes faster [87]. This also contradicts with the study carried on M. oleifera, showed that the application of different PEG treatments resulted in significant differences in germination rate [85].

Regarding germination kinetics, the results indicated that water stress caused acceleration and a high germination rate of M. stenopetala seeds. This is because increasing the concentration of PEG improves the germination movement of Moringa seeds by stimulating the secretion of plant hormones such as auxins and cytokinins, which help regulate seed growth and improve water stress response [88,89]. The mean germination time decreased with increasing PEG treatments, and the analysis of variance associated with the mean germination time showed that water potential and interaction had a significant effect which contradicts to other studies for soybean and blackgram [90]. Treatments have been shown to dramatically accelerate mean germination time, even under highly demanding conditions [91,92].

Analysis of the results also showed that high PEG treatments positively influence the germination index [93,94]. The application of a high concentration of PEG leads to a rapid and significant germination index, while the lowest concentration contains a limited amount of nutrients or active compounds that do not improve seed germination [67,74]. These results contrast on soybean and blackgram, which confirmed that the highest germination index was obtained with the lowest concentration of PEG [95,96]. Also, the results obtained show that M. stenopetala exhibits notable tolerance to PEG-induced water stress. Contrary to responses generally observed in other species, such as M. oleifera [85], where water stress leads to a reduction in growth and biomass, our observations indicate a significant increase in aerial and root development with increasing PEG concentrations (4–12 g/L). This growth could be linked to a moderate lowering of osmotic pressure, promoting water uptake by roots and thus stimulating vegetative growth [97].

Although root growth is often compromised under stress [98], M. stenopetala seems to mobilize adaptive mechanisms to maintain or even improve its biomass. This ability is also illustrated by the increase in the root/aerial ratio, indicating an advantageous distribution of resources. Furthermore, the significant reduction in leaf area observed in the face of water stress is a classic water-saving strategy [99]. This adaptation is reinforced by the partial closure of stomata [63], limiting transpiration while maintaining photosynthetic activity, as suggested by the rise in chlorophyll a content.

Analysis of chlorophyll pigments reveals relative stability of chlorophyll b and total chlorophyll (a + b), while chlorophyll a increases significantly under stress. This response could result from increased concentration due to reduced leaf cell size [100,101], reflecting efficient photosynthetic adaptation. In contrast, proline, often considered a marker of water stress [102]. showed a significant decrease, suggesting that M. stenopetala does not systematically resort to this osmotic adjustment mechanism. This response contrasts with many species where proline accumulation correlates with stress severity [103–105]. Conversely, soluble sugars increased with stress intensity, consistent with their recognized role in protecting cellular structures and maintaining metabolic functions [106–108]. The rise in free amino acids observed in aerial and root parts (PEG12%) also testifies to a metabolic response to stress, probably linked to an intensification of proteolysis and a reduction in protein synthesis [109,110]. In sum, the results highlight the remarkable ability of M. stenopetala to tolerate moderate to severe water deficit conditions, mobilizing distinct and compelling morpho-physiological and biochemical mechanisms, making it a promising species for arid zones.

The measured drought indices based on PEG induced drought treatments indicate that Moringa plant has various response to abiotic stress. The PEG 8% treatment showed high results in GSI, MPI, STI, and HM which reflect a significant enhancement compared to control treatment [111,112]. This suggests that moderate stress may promote physiological mechanisms that improve plant biomass accumulation and tolerance of abiotic [79]. The increased GSI and STI indices at 8% PEG prove the ability of moringa seedlings to maintain growth and development as under non-stressed conditions [113,114]. This finding indicates that moringa can serve as a drought-tolerant crop and highlights the importance of stress dose optimization to achieve seedlings hardening before transplanting.

## 5. Conclusion

Moringa stenopetala demonstrated strong adaptive responses to PEG-induced drought stress during its early growth. Under moderate stress (PEG 8%), the seedlings of Moringa stenopetala showed improved stem and root development, maintained high germination rates, and exhibited effective biochemical adjustments through changes in proline and sugar levels. This study also lays the groundwork for developing screening tools using drought indices and physiological traits. Moving forward, research should expand to include multiple genotypes, molecular analyses, and field trials to identify the most promising lines of Moringa stenopetala and confirm their performance under field-drought conditions

## Supporting information

S1 Table Descriptive statistics of the studied parameters. (DOCX)

S2 Table Drought Indices that extracted from the obtained data. (DOCX)

S1 File Inclusivity-in-global-research-questionnaire. (DOCX)
