# Supplementary material for: Performance and tolerance of Moringa stenopetala exposed to drought stress during germination and growth
Source: PLoS One. 2025 Nov 3;20(11):e0329544. doi: 10.1371/journal.pone.0329544 (PMC12582485; doi:10.1371/journal.pone.0329544)
Supplement: S1 Table — (DOCX) [file pone.0329544.s001.docx]

**Performance and Tolerance of Moringa stenopetala Exposed to Drought Stress During Germination and Growth**

**Supplementary data**

| **S1-Table. Descriptive statistics of the studied parameters** | | | |
| --- | --- | --- | --- |
| Variable | PEG | Chl a | StDev |
| ChlA | Control | 11.22 | 1.552 |
|  | PEG 4% | 21.19 | 2.25 |
|  | PEG 8% | 21.293 | 1.002 |
|  | PEG 12% | 19.35 | 2.01 |
|  | PEG | Chl b |  |
| ChlB | Control | 19.583 | 0.576 |
|  | PEG 4% | 11.02 | 1.12 |
|  | PEG 8% | 9.057 | 0.9358 |
|  | PEG 12% | 7.453 | 0.8457 |
|  |  | Total Chl |  |
| Total Chl | Control | 30.8 | 2.06 |
|  | PEG 4% | 32.21 | 2.279 |
|  | PEG 8% | 30.35 | 1.36 |
|  | PEG 12% | 26.8 | 2.23 |
|  |  | Chl ratio |  |
| Chl ratio | Control | 0.5719 | 0.0749 |
|  | PEG 4% | 1.947 | 0.1144 |
|  | PEG 8% | 2.3507 | 0.181 |
|  | PEG 12% | 2.598 | 0.106 |
|  |  | Girmination Rate |  |
| Girmination Rate | Control | 99.5 | 3.88 |
|  | PEG 4% | 95.333 | 2.041 |
|  | PEG 8% | 97.143 | 3.388 |
|  | PEG 12% | 82.5 | 2 |
|  |  | Germination speed |  |
| Germination speed | Control | 15 | 1.3 |
|  | PEG 4% | 26 | 1.5 |
|  | PEG 8% | 87 | 2.5 |
|  | PEG 12% | 92.73 | 4.51 |
|  |  | MGT (days) |  |
| MGT (days) | Control | 99.1 | 3.5 |
|  | PEG 4% | 87 | 1.794 |
|  | PEG 8% | 73.2 | 2 |
|  | PEG 12% | 66.067 | 2.503 |
|  |  | GVI |  |
| GVI | Control | 55 | 2.35 |
|  | PEG 4% | 59.67 | 2.15 |
|  | PEG 8% | 91.2 | 2.35 |
|  | PEG 12% | 80.15 | 2.08 |
|  |  | GI |  |
| GI | Control | 18.03 | 2.75025 |
|  | PEG 4% | 30.6 | 3.34 |
|  | PEG 8% | 102.1 | 5.15 |
|  | PEG 12% | 93.03 | 3.95 |
|  |  | Proline |  |
| Proline | Control | 0.3333 | 0.0465 |
|  | PEG 4% | 0.3417 | 0.0582 |
|  | PEG 8% | 0.4873 | 0.046912 |
|  | PEG 12% | 0.5927 | 0.040811 |
|  |  | Soluble suger in stem |  |
| Soluble suger in stem | Control | 0.41133 | 0.03531 |
|  | PEG 4% | 0.4623 | 0.0366 |
|  | PEG 8% | 0.4897 | 0.044615 |
|  | PEG 12% | 0.4937 | 0.04672 |
|  |  | Soluble suger in root |  |
| Soluble suger in root | Control | 0.2627 | 0.01344 |
|  | PEG 4% | 0.2897 | 0.012495 |
|  | PEG 8% | 0.2947 | 0.0182 |
|  | PEG 12% | 0.285 | 0.011433 |
|  |  | Amino acid in stem |  |
| Amino acid in stem | Control | 2.4 | 0.086 |
|  | PEG 4% | 2.524 | 0.0509 |
|  | PEG 8% | 2.678 | 0.04111 |
|  | PEG 12% | 3.138 | 0.0605 |
|  |  | Amino acid in root |  |
| Amino acid in root | Control | 1.011 | 0.211 |
|  | PEG 4% | 3.441 | 0.566 |
|  | PEG 8% | 2.106 | 0.348 |
|  | PEG 12% | 3.662 | 0.3827 |
| *Four different concentrations of Poly Ethylene Glycol (PEG-6000) solution were applied on Moringa seeds to study the impact of drought stress on its germination and growth (Control, PEG4%, PEG8%, and PEG12%)* | | | |
