# Supplementary material for: Performance and tolerance of Moringa stenopetala exposed to drought stress during germination and growth
Source: PLoS One. 2025 Nov 3;20(11):e0329544. doi: 10.1371/journal.pone.0329544 (PMC12582485; doi:10.1371/journal.pone.0329544)
Supplement: S2 Table — (DOCX) [file pone.0329544.s002.docx]

**Performance and Tolerance of Moringa stenopetala Exposed to Drought Stress During Germination and Growth**

**Supplementary data**

| **S2-Table. Drought Indices that extracted from the obtained data** | | | | | |
| --- | --- | --- | --- | --- | --- |
| Treatment | GSI (Ys/Yp) | TOL (Yp−Ys) | MPI (0.5×(Ys+Yp)) | STI ((Ys×Yp)/Yp) | HM ((2×Ys×Yp)/(Ys+Yp)) |
| PEG 4% | 1.18125 | -0.145 | 0.8725 | 0.905 | 0.8485 |
| PEG 8% | 1.35 | -0.28 | 0.94 | 1.3325 | 0.938 |
| PEG 12% | 0.93125 | 0.055 | 0.7725 | 0.735 | 0.774 |
| GSI = Growth Stability Index; TOL = Tolerance; MPI = Mean Productivity Index; STI = Stress Tolerance Index, HM = Harmonic Mean; Ys = accumulated weight under drought stress; Yp = accumulated weight under non-stress (control) conditions. | | | | | |
